# Engineering of impact ionization characteristics in In_0.53_Ga_0.47_As/Al_0.48_In_0.52_As superlattice avalanche photodiodes on InP substrate

**DOI:** 10.1038/s41598-020-73810-w

**Published:** 2020-10-07

**Authors:** S. Lee, M. Winslow, C. H. Grein, S. H. Kodati, A. H. Jones, D. R. Fink, P Das, M. M. Hayat, T. J. Ronningen, J. C. Campbell, S. Krishna

**Affiliations:** 1 Department of Electrical and Computer Engineering, The Ohio State University, Columbus, OH 43210 USA; 2 Department of Physics, University of Illinois, Chicago, IL 60607 USA; 3 Department of Electrical and Computer Engineering, University of Virginia, Charlottesville, VA 22904 USA; 4 Department of Electrical and Computer Engineering, Marquette University, Milwaukee, WI 53233 USA

**Keywords:** Electrical and electronic engineering, Photonic devices

## Abstract

We report on engineering impact ionization characteristics of In_0.53_Ga_0.47_As/Al_0.48_In_0.52_As superlattice avalanche photodiodes (InGaAs/AlInAs SL APDs) on InP substrate to design and demonstrate an APD with low *k*-value. We design InGaAs/AlInAs SL APDs with three different SL periods (4 ML, 6 ML, and 8 ML) to achieve the same composition as Al_0.4_Ga_0.07_In_0.53_As quaternary random alloy (RA). The simulated results of an RA and the three SLs predict that the SLs have lower *k*-values than the RA because the electrons can readily reach their threshold energy for impact ionization while the holes experience the multiple valence minibands scattering. The shorter period of SL shows the lower *k*-value. To support the theoretical prediction, the designed 6 ML and 8 ML SLs are experimentally demonstrated. The 8 ML SL shows *k*-value of 0.22, which is lower than the *k*-value of the RA. The 6 ML SL exhibits even lower *k*-value than the 8 ML SL, indicating that the shorter period of the SL, the lower *k*-value as predicted. This work is a theoretical modeling and experimental demonstration of engineering avalanche characteristics in InGaAs/AlInAs SLs and would assist one to design the SLs with improved performance for various SWIR APD application.

## Introduction

Avalanche photodiodes (APDs) have been widely used for various short-wavelength infrared (SWIR, 1.5–3 microns) applications such as optical communication^1^, 3D imaging^2^, single-photon detection^3^ and LIDAR^4^. APDs are preferred for these applications, relative to conventional p-i-n PDs, because of the internal multiplication gain. This gain significantly improves the sensitivity of a sensing and imaging system when the circuit noise limits the signal to noise ratio (SNR). Therefore, APDs allow one to detect a lower signal magnitude coming through any harsh environment that degrades the initial intensity of the input signal.

There is interest in APDs operating at 1500 nm or 2000 nm for LIDAR active sensing and imaging applications. The three critical characteristics of an APD are the multiplication gain (*M*), the dark current (*I*_*d*_)_,_ and the excess noise factor (*F*(*M*)). When a high electric field is applied to the materials, carriers (electrons and holes) can gain enough energy to impact ionize, resulting in *M*. However, this high electric field gives rise to large tunneling dark current due to relatively narrow bandgap and high excess noise caused by a stochastic process of multiplication events in the materials. This problem can be mitigated by using separate, absorption, charge, and multiplication (SACM) APDs, where a high electric field is located at a wide bandgap multiplication layer (M-layer) while a low electric field exists in a narrow bandgap absorption layer. To prevent strain-induced defects that reduce the device performance, SACM APDs need to be made from material layers that are lattice-matched to the host substrate. InP substrates are commonly used for commercial APD technologies because they are reasonably cheap and commercially available up to 4″ in diameter. Since good absorbers such as In_0.53_Ga_0.47_As (1550 nm)^5^ and In_0.53_Ga_0.47_As/GaAs_0.51_Sb_0.49_ (2000 nm)^6^ on InP substrates are already well-developed, they can easily be combined with wide bandgap materials used for the M-layer such as InP^7^, Al_0.48_In_0.52_As^8^, Al_x_Ga_1−x_As_0.56_Sb_0.44_^9^ and AlAs_0.56_Sb_0.44_^10^, potentially realizing high-performance SACM APDs.

To minimize excess noise in an M-layer, there must be a large difference between the impact ionization coefficients for electrons (*α*) and holes (*β*). The excess noise can be quantified as *F*(*M*), and is estimated by McIntyre’s local field theory^11^, *F*(*M*) = *kM* + (1 − *k*) [2–1/*M*]. Here, *k* is the ratio of *α* and *β* impact ionization coefficients, specifically *k* = *β*/*α* when *β* > *α* and *k* = *α*/*β* when *β* < *α,* by convention. It should be noted that when *k*-value is low, *F*(*M*) increases slowly as *M* increases. Thus, a low *k*-value is desired to make an APD highly sensitive. A low *k*-value in an M-layer can be achieved simply by selecting a material system that has favorable *α* or *β*. For instance, Si has a *k*-value of < 0.05^12^, and InAs^13^ and HgCdTe^14^ have *k*-values of 0. There has been active research to find suitable materials for the M-layer that are lattice-matched to InP substrates. Tan and David et al. have developed thin Al_x_Ga_1−x_As_0.56_Sb_0.44_ APDs (hereafter AlGaAsSb) on InP substrates^15^. A 100 nm thick M-layer provided with a *k*-value of 0.1 with the assistance of dead space. However, an APD technology at least needs to be comparable to Si APDs, and the AlGaAsSb APDs cannot achieve such a low *k*-value with relatively thin layers. A way to attain the low *k*-value is to have an M-layer that is ~ 1500 nm thick^16^. Unfortunately, AlGaAsSb APDs cannot be randomly grown with such large thicknesses due to the existence of a large miscibility gap, causing phase separation during the growth.

Recently, the superlattice (SL) or digital alloy (DA) growth technique has opened up a new path to growing materials for APDs and has unlocked a new means for reducing the *k*-value of an APD. The terms SL and DA have been used interchangeably in the APD literature. One monolayer (ML) refers to half the lattice constant along the (100) direction for a zinc blende structure. We use the term SL in this paper to refer to periodic In_0.53_Ga_0.47_As/Al_0.48_In_0.52_As heterostructures, and specifically consider periods of three values (4 ML, 6 ML, and 8 ML). The limiting case of a 2 ML SL is a random alloy. The important point is that engineering the periodicity of the lattice has a dramatic impact on the band structure and the transport of carriers. Campbell and Bank et al. have demonstrated an 890 nm thick Al_x_In_1−x_As_y_Sb_1−y_ SL grown on a GaSb substrate using four binary layers in a period as an alternative to the bulk random alloy (RA)^17^. Their Al_0.7_In_0.3_As_0.3_Sb_0.7_ APD showed a *k*-value of 0.01, which is comparable to Si. Thick AlAs_0.56_Sb_0.44_ APDs on InP substrates have also been successfully grown as AlAs/AlSb SLs for a 1550 nm thick M-layer. The SLs exhibited an extremely low *k*-value of 0.005^16^. To investigate the origin of lower *k*-value in a SL APD, AlAs/InAs SL (compositionally the same as Al_0.48_In_0.52_As RA) APDs were grown on an InP substrate. They achieved a lower *k*-value (*k* = 0.03) than an Al_0.48_In_0.52_As RA (*k* = 0.24)^18^. The origin was investigated in terms of electronic band structures of the SL and RA APDs. It was found that a mini gap exists in the hole bands of the SL which inhibits the holes from gaining enough energy to impact ionization while electrons can easily reach their threshold energy for impact ionization. This demonstrates that the advantage of using an SL for the M-layer is that its electronic structure can be engineered to realize a low *k*-value. In other words, one can modify material properties such as effective masses, band gaps (*E*_*g*_), *α*, and *β* with various thicknesses of the alternating layers of the SLs^19^. For example, Grein, Ghosh and Krishna have demonstrated electron-APD and hole-APD with InAs/GaSb type-II SLs for mid-IR applications, specifically an extremely low F(M) of 0.8–1.2 and *k* ~ 0^20,21^. This indicates that if the electronic structure of an SL is properly designed, one can favorably achieve a high-performance APD.

The Al_x_Ga_y_In_1−x−y_As (hereafter AlGaInAs) quaternary alloy has proven to be a useful material system for optoelectronic devices. For APDs, AlGaInAs alloys have only been exploited for grading and charge layers in SACM APDs, since they can be designed with bandgaps intermediate between the absorber and multiplication layers^8–10^. This material has not been used solely as an M-layer since APDs with AlGaInAs RAs have a relatively high *k*-value of between 0.24 (Al_0.48_In_0.52_As)^22^ and 0.5 (In_0.53_Ga_0.47_As)^23^. A 1000 nm thick Al_0.24_Ga_0.24_In_0.52_As RA showed a *k*-value of ~ 0.5^24^. However, In_0.53_Ga_0.47_As/Al_0.48_In_0.52_As (hereafter InGaAs/AlInAs) heterostructure APDs have demonstrated a promising *k*-value of 0.05 due to the larger conduction band offset than the valence band offset between Al_0.48_In_0.52_As and In_0.47_Ga_0.53_As^25^. Therefore, InGaAs/AlInAs SL may have the potential to achieve a reduced *k*-value. Also, an InGaAs/AlInAs SL facilitates more straightforward material growth of SACM APDs with an In_0.53_Ga_0.47_As absorber.

In this work, we discuss theoretical modeling and experimental demonstrations of a p-i-n configuration of InGaAs/AlInAs SLs as M-layer candidates. The thicknesses of constituent layers in the SL were designed to have the same average alloy composition as Al_0.4_Ga_0.07_In_0.53_As RA. The SL periods were chosen to be 4 ML, 6 ML, and 8 ML. As our first step, we computed the electronic band structures and impact ionization coefficients for those SL periods. These results were compared with the compositionally similar RA and analyzed in terms of avalanche threshold energies and scattering mechanism for electrons and holes. We experimentally demonstrate the SLs with periods of 6 ML and 8 ML and show the theoretical predictions are in good agreement with the experimental results.

## Results

The entire theoretical computations were done at 240 K while experimental demonstrations were performed at 300 K. The reason is that at low temperatures, band engineering features are more pronounced because thermal energies restrict carriers to relatively few minibands. The difference between 240 and 300 K will not create significant inconsistency between theory and experiment.

### Simulation for band structure engineering

**Figure 1:**
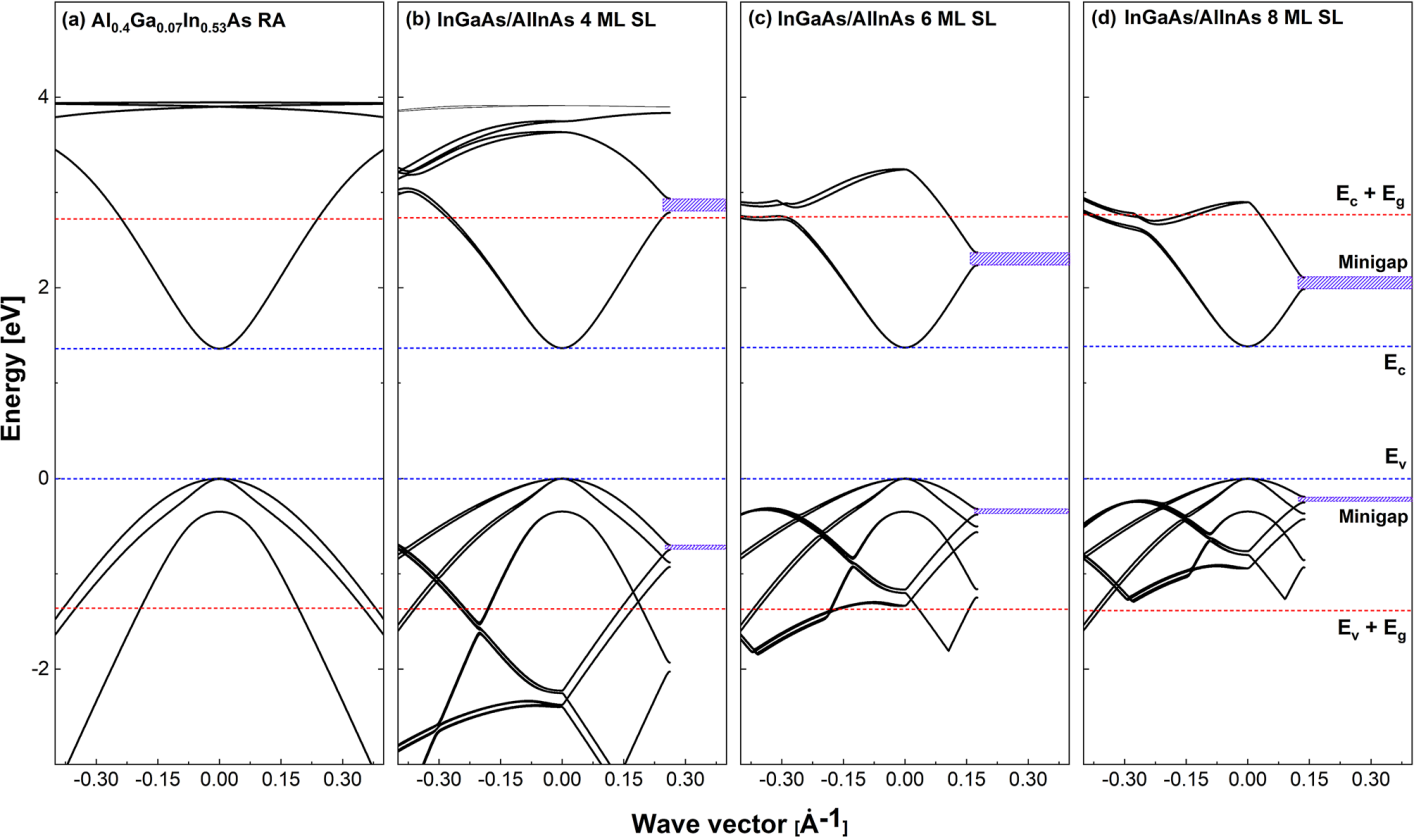
(**a**) Computed electronic structures at 240 K of Al_0.4_Ga_0.07_In_0.53_As RA, (**b**) the 4 ML InGaAs/AlInAs SL, (**c**) the 6 ML InGaAs/AlInAs SL, and (**d**) the 8 ML InGaAs/AlInAs SL.

A realistic and reliable SL electronic band structure is the principal input to evaluate impact ionization rates. Our methodology follows that of ref.^19^, which also contains the employed input parameters and is summarized here for completeness. The SL electronic band structures were calculated employing a 14-band SL **K.p** method^26^. It should be noted that the reliability of the SL **K.p** method becomes weaker as the wavevector deviates from the zone-center. Therefore, the SL **K.p** approach should be considered as the source of a physical explanation with the understanding that the quantitative predictions are approximate. The calculations use a heterostructure restricted basis formalism non-perturbatively employing fourteen bulk bands^27^. The heterostructure multiband Hamiltonian is then transformed into Fourier space and diagonalized. An envelope function formalism^28^ is used to obtain the zone-center SL states. States throughout the zone are then evaluated using the SL **K.p** method^29^. The highly non-parabolic band structures and momentum matrix elements computed employing the 14-band SL **K.p** method are used directly with a mesh spacing of 0.002 Å^−1^ as input for the evaluation of the impact ionization rates of hot holes and electrons. The designs of RA and the three SLs appear in Table 1, together with bandgaps (*E*_*g*_) extracted from the electronic structures of all four materials. Their computed electronic structures at 240 K appear in Fig. 1.

**Table 1:** Layer thicknesses for the three SLs, and band gaps at 240 K as predicted by the electronic structure calculations for all four structures.

SL periods	Thickness [nm] (Al_0.52_In_0.48_As)	Thickness [nm] (In_0.53_Ga_0.47_As)	*E*_*g*_ [eV]
RA	–	–	1.360
4 ML	1.02	0.18	1.367
6 ML	1.53	0.27	1.372
8 ML	1.96	0.34	1.384

The electronic band structures of the SLs were designed to optimize the electron-initiated to hole-initiated impact ionization coefficient ratio. Specifically, the conduction minibands (*E*_*c*_) were engineered to promote the impact ionization of hot electrons, whereas valence minibands (*E*_*v*_) were designed to suppress the ionization of hot holes. This is accomplished through choosing the SL period to permit electrons to impact ionize from the lowest conduction miniband, but holes must scatter among multiple valence minibands to reach their impact ionization threshold. Transport inhibited by zone folding reduces hole-initiated impact ionization while electrons undergo impact ionization similar to that of a bulk material. This can be seen from the band structure appearing in Fig. 1. The Al_0.4_Ga_0.07_In_0.53_As RA has its lowest conduction, and the highest valence bands that cross line an energy gap above the conduction band minimum (upper dotted red line) and an energy gap below the valence band maximum (lower dotted red line), respectively. These lines represent minimum impact ionization threshold energies. This contrasts with the 4 ML SL where the lowest conduction miniband just crosses the line, suggesting the shortest period 4 ML SL is superior in terms of the electron to hole impact ionization coefficient ratio differing from one. The 6 ML and 8 ML SLs do not exhibit these desirable band-structure characteristics. However, they still show electron-initiated APD behavior due to their much heavier hole masses compared to the RA and multiple valence bands scattering for holes.

### Monte Carlo simulation

**Figure 2:**
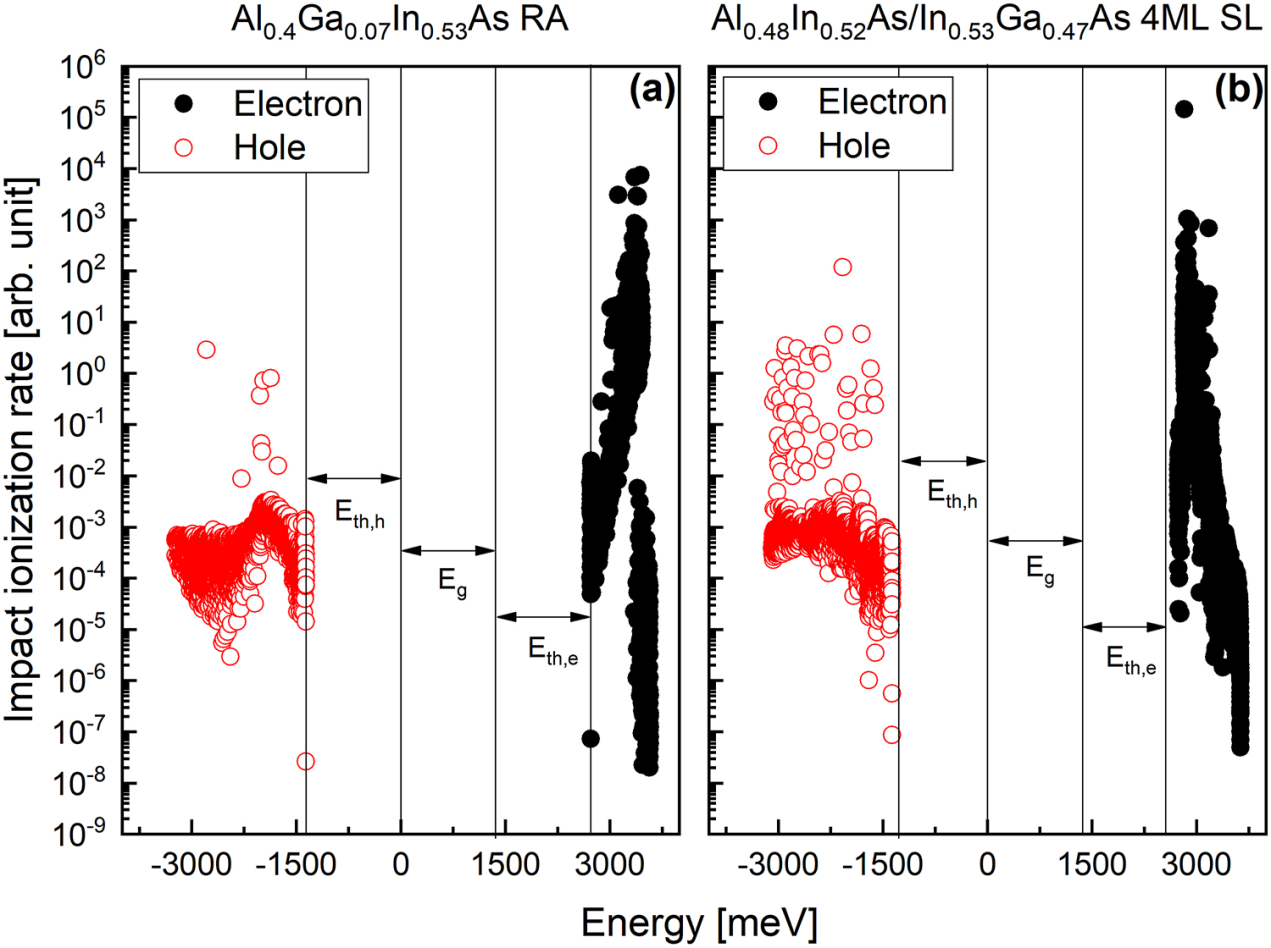
(**a**) 240 K computed ionization rates of the Al_0.4_Ga_0.07_In_0.53_As RA and the 4 ML InGaAs/AlInAs SL (**b**). Hot holes are marked in red, and electrons in black. Both show hot electron (*E*_*th,e*_) and hole (*E*_*th,g*_) threshold energies nearly equal to the energy gap (*E*_*g*_).

The formalism used to compute the impact ionization rates follows that of ref.^19^, and is summarized here for completeness. The total impact ionization rate is obtained from Fermi’s Golden Rule. **K**-space integrals are evaluated with an adaptive mesh Monte Carlo algorithm. The matrix elements are written in terms of a Slater determinant of SL states obtained from the electronic structure calculations. The Coulomb potential is screened by the Debye screening length. Umklapp processes are included in the summations over the SL reciprocal lattice vectors. First-order SL **K.p** perturbation theory is employed to evaluate the overlaps of the SL wave functions at different points in **K**-space. This permits the square of the screened Coulomb matrix element to be expressed in terms of SL momentum matrix elements, also obtained from the electronic structure calculations. The wave vector-dependent impact ionization rate is averaged over the entire Brillouin zone (BZ) and set of minibands to yield the impact ionization rate as a function of hot carrier energy. Figure 2 plots the 240 K computed ionization rates of the Al_0.4_Ga_0.07_In_0.53_As RA and the 4 ML SL. It should be noted that the plots were obtained under weak field conditions. Both have threshold energies of electrons (*E*_*th,e*_) and hole (*E*_*th,h*_) nearly equal to the energy gap. However, somewhat promising, the electron-initiated rate of the 4 ML SL has a stronger onset than that of the RA, and the hole-initiated rate of the 4 ML SL is overall lower than that of the RA. This promotes electron-initiated impact ionization over that of holes. The 6 ML and 8 ML SLs were not examined here since they are seen as possessing electronic structure features intermediate between the extremes of the bulk alloy and 4 ML SL.

**Figure 3:**
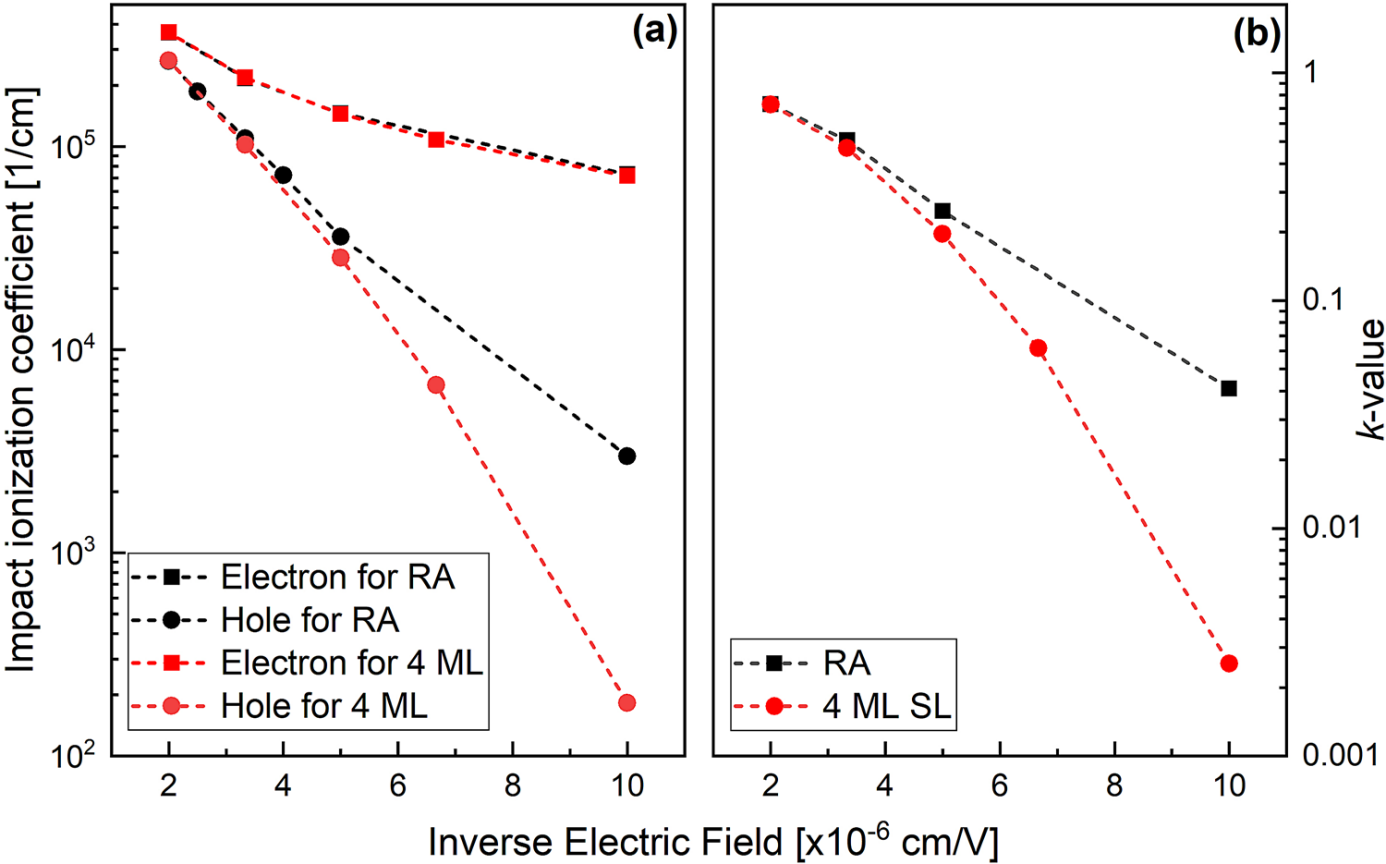
(**a**) Simulated impact ionization coefficients for RA and the 4 ML SL and (**b**) extracted *k*-value as a function of inverse electric field.

Figure 3a shows computed electron- and hole-initiated impact ionization coefficients for the RA and the 4 ML SL simulated via an ensemble Monte Carlo transport kernel^30–32^. The $\alpha$ is almost the same for both the RA and the 4 ML SL, while the $\beta$ of the 4 ML SL is lower than that of the RA in the electric field range from 100 to 300 kV/cm. As a result, the *k*-value of the 4 ML SL is lower than that of the RA, and the difference of their *k*-value becomes much more significant at the lower electric field region, as shown in Fig. 3b.

## Experimental demonstration

In this section, we will experimentally demonstrate the APDs designed in previous section. The 4 ML SL is not considered here due to the limited equipment available for the difficulty of achieving low background doping. However, the 6 ML and 8 ML SLs are realized and appeared to be enough for the demonstration of the physical prediction discussed in section I. All measurements were performed at 300 K while computations were carried out at 240 K.

Capacitance–voltage (C–V) measurements were taken at a probe station using specialized test structures. These circular test structures were blind (had no aperture) and had a diameter of 300 µm, much larger than the photodiodes. The large area increases the device capacitance, making it easier to measure. The ratio of the junction area and surface area is proportional to the device’s diameter, so larger devices minimize surface effects. Phase corrections and open circuit corrections were taken prior to measurements^33^.

**Figure 4:**
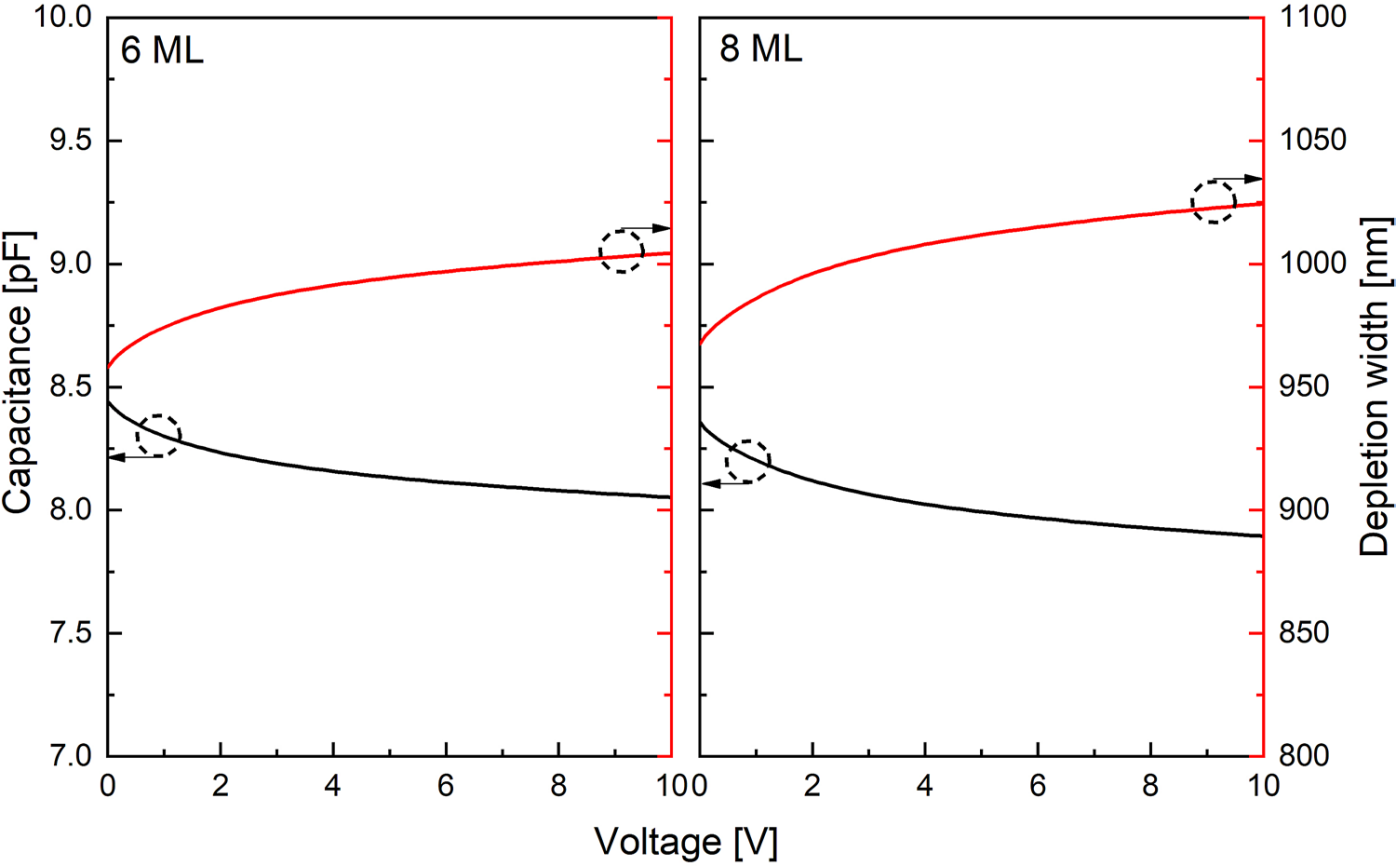
The result of C–V measurements for 6 ML and 8 ML InGaAs/AlInAs SL APDs.

C-V data at room temperature is shown in Fig. 4 for the 6 ML and 8 ML SLs. Both show that the depletion width is approximately 1 µm when fully depleted, as expected. The background doping concentration, *n*_*bg*_, was calculated to be < 1 $\times$ 10^16^ cm^−3^, indicating low residual impurities in the SL materials.

Current–voltage (I–V) characteristics were measured with a Keithley 2400 source meter. A 543 nm He–Ne laser was used as an optical source to illuminate the devices. To calculate *M*, we needed to determine the unity gain point of the 6 ML and 8 ML APDs. For a punch-through APD such as a SACM APD, it is not straightforward to determine the unity gain point because the curves of C–V and I–V under illumination are not saturated at a bias voltage. However, for a PIN APD the unity gain point was easily deduced due to the flat region of the I–V curve under illumination and the saturated region of the C–V curve. The devices are shown to be fully depleted at −10 V as shown in Fig. 4. Therefore, this bias was chosen as the unity gain voltage.

**Figure 5:**
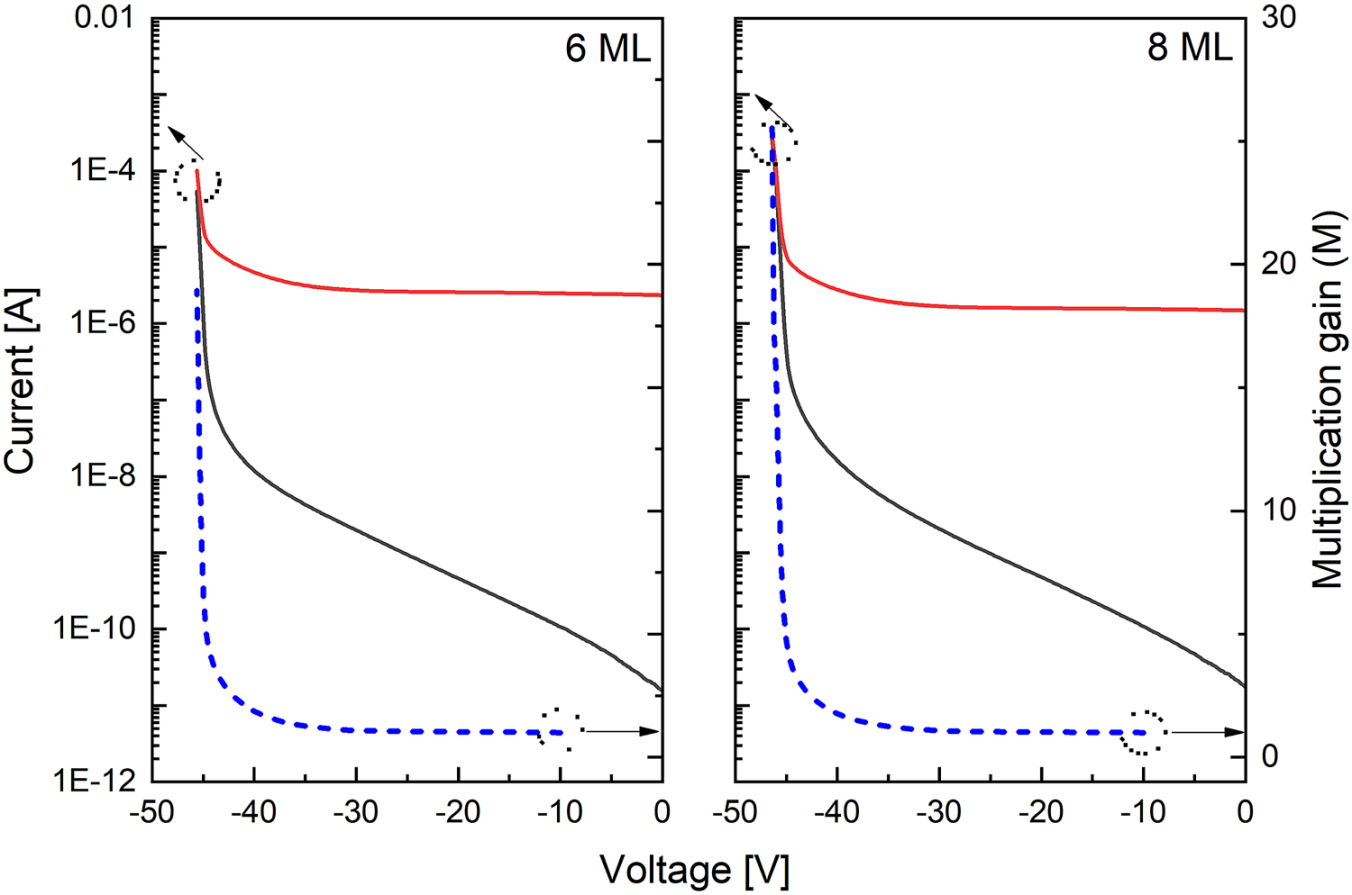
I–V characteristics for 6 ML (left) and 8 ML (right). The black and red solid lines indicate dark current and current under illumination, respectively. The dashed blue line is calculated gain from two solid line curves.

The I–V data for the 6 ML and 8 ML SLs are shown in Fig. 5. Both have breakdown voltages around −44 V. The maximum *M* of the 6 ML SL approached 20, but was over 25 for the 8 ML SL. The catastrophic surface breakdown which destroyed the devices occurred at slightly different biases for each device, causing their maximum *M* to differ. Thus, the small difference in the maximum *M* measured between the 6 ML and 8 ML SLs is not a conclusive result.

**Figure 6:**
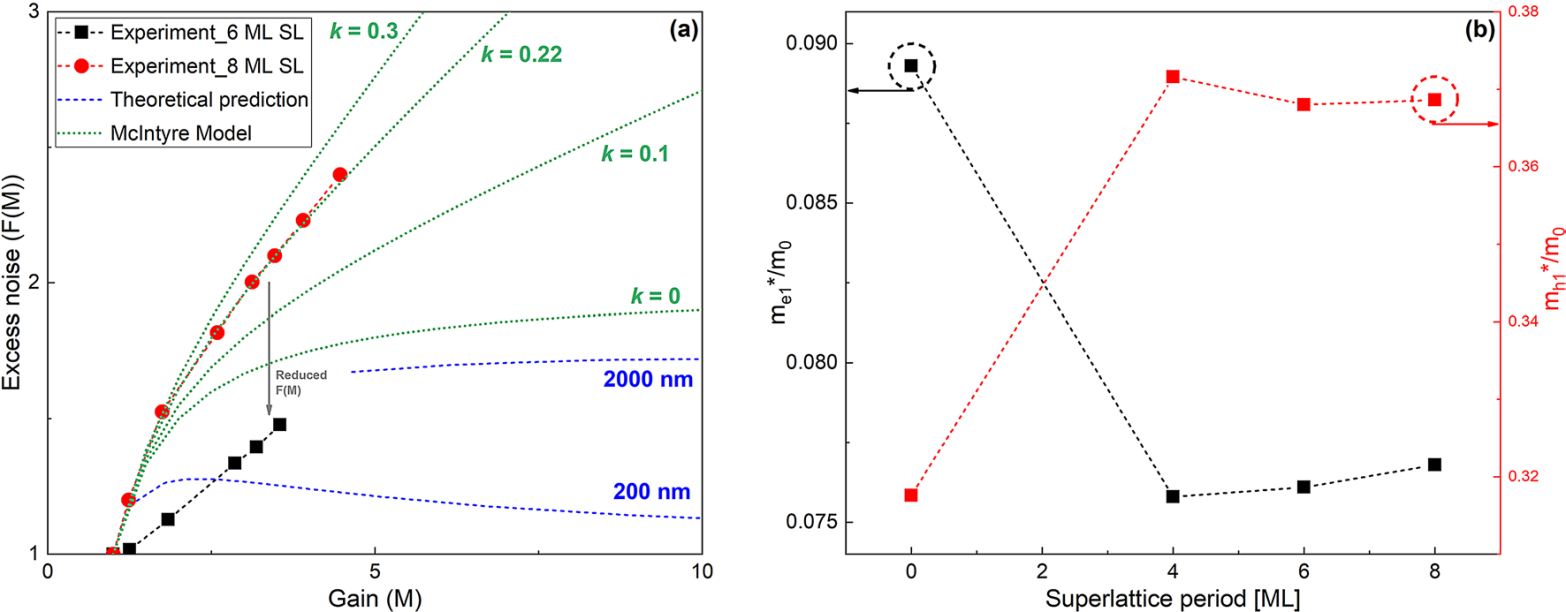
(**a**) McIntyre model cases (green), dead space predictions when *k* = 0 (blue), and experimentally observed *F(M*) (dotted black and red) for InGaAs/AlInAs SLs. (**b**) The effective masses of electron and hole as a function of SL period. The effective masses were extracted by parabolic approximation around the zone center.

The results of the excess noise measurement are shown in Fig. 6a. Even though the gain values are small, the *F*(*M*) of the 6 ML SL drops below *F*(*M*) = 2 (*k*-value = 0, McIntyre limit), unlike the 8ML SL. This indicates that the gain is deterministic in nature. In order to understand the non-local effects, we undertook the modeling of the dead space for two different multiplication layer thicknesses (200 nm and 2000 nm) as shown by the dotted blue lines. The electron’s dead space is calculated by dividing the electron ionization threshold energy by the electric field^34^. The electron ionization coefficients for AlGaInAs are obtained from Tsuji et al^24^. This shows that non-local effects are occurring in the sample with 6 ML SL, and thus deterministic multiplication event. This needs further investigation, especially when the gain is increased. The current tolerance of the analyzer prevented *F*(*M*) measurements at higher gains, indicated in Fig. 6a. When fitted to McIntyre’s model^11^, the *k*-value of the 8 ML SL was a close match to ~ 0.22. This value is lower than what has been previously reported for Al_0.48_In_0.52_As RA and Al_0.24_Ga_0.24_In_0.52_As RA^24^, indicating growing this material as a digital alloy reduces the excess noise. The 6 ML SL data does not fit to McIntyre’s model but shows significantly lower excess noise than the 8 ML SL.

## Discussion

We modeled one Al_0.4_Ga_0.07_In_0.53_As RA and three InGaAs/AlInAs SLs (4 ML, 6 ML, and 8 ML) and briefly analyzed their electronic structures. The SLs were also experimentally realized. Here, more details on engineering impact ionization characteristics are discussed in terms of material and device perspectives in the SLs.

Interestingly, all SLs have almost the same *E*_*g*_, as described in Table 1, due to the weak quantum confinement in the thin InGaAs well layer. This results in nearly the same *E*_*th*_ for the three SLs as ~ *E*_*g*_. Due to the SL periodicity, the 1st BZ edge of the SLs in the growth-axis direction becomes very short compared with the RA. The 1st BZ width of the SLs reduces as the period of the SL increases, as shown in Fig. 1. The optimal design for an electron SL, such as the 4 ML SL illustrated in Fig. 1b, will allow electrons to reach their *E*_*th,e*_ from the lowest conduction miniband while holes must scatter between multiple minibands before reaching their threshold. The effective masses of the electron (${m}_{e}^{*}$) and hole (${m}_{h}^{*}$) for the SLs were extracted from the computed band structures, and their ratios to the free electron mass (*m*_*0*_) are plotted in Fig. 6b. All SLs have similar effective masses for both electron and holes, and lower electron and higher hole effective masses than the RA. The effective masses in the SLs help the APDs to have low-*k* values because the relatively light electrons can more readily reach their threshold energy than holes. The 6 ML and 8 ML SLs are less desirable for high-performance APDs than the 4 ML one because the electron energy at 1^st^ BZ edge lies below *E*_*th,e*_. However, we observed in Fig. 6a that the 6 ML and 8 ML SLs still showed a lower *k*-value than the RA, although they do not satisfy the criteria of an optimal SL design. We here consider another design factor for the SLs, the presence of mini gaps. In Fig. 1d, there are mini gaps at the upper energy edge of *E*_c_ and the lower energy edge of *E*_*v*_ in the growth-direction (these mini gaps are present in all the SLs). The mini gaps are important for carrier transport in the SLs because these energies will either provide hot carriers with a bridge to tunnel to or with an obstacle to prevent them from tunneling to another higher miniband^35^. The mini gaps are mostly determined by the band offset between two alternating layers and the strength of the interaction between adjacent energy states to perturb each other. A zero band offset either at conduction or valence band is highly preferred^35^. In the case of the SLs, the mini gaps are not so large that they do not prevent electrons and holes from tunneling to the higher band energies, thus reaching impact ionization threshold. At the large applied electric field, the tunneling probability even increases further^36,37^. We note that mini gaps can be a massive hindrance to the hole due to the larger ${m}_{h}^{*}$ in the SLs, even though the hole’s mini gaps are smaller than the electron’s. As a result, electron-initiated impact ionization can still occur in the 6 ML and 8 ML SLs, resulting in low *k*-value.

From the perspective of material design, the best SL at a given *E*_*g*_ can originate from three design factors; the 1st BZ edge along the growth direction, the effective masses, and the mini gaps, as discussed. Therefore, in order to make a high-performance SL, the selection of constituent materials and the thickness of their layers are very crucial. From the perspective of device design, we can take advantage of thick or thin M-layers. For a thin M-layer that is submicron, the impact ionization characteristics of carriers are highly influenced by the history of carrier transport due to the presence of the dead space, which is the distance where carriers need to travel before impact ionization^38^. In this situation, the probability density function of impact ionization becomes more contracted, and the ionization more predictable, resulting in a highly deterministic impact ionization event. Therefore, *F*(*M*) can be lower than 2 and McIntyre’s local field theory no longer governs such an APD. For a thick M-layer, we also have the opportunity to achieve a low *k*-value at a low electric field region^39^. Such a single carrier-initiated APD has a large difference between *α* and *β* at a low electric field, approaching a *k*-value of 0. Therefore, if the thickness of the M-layer is thick enough for inducing avalanche breakdown before the *k*-value significantly increases, we can achieve an APD with *F*(*M*) < 2. The 6 ML SL shows *F*(*M*) < 2, which is the limit of *F*(*M*) in McIntyre’s theory. It indicates that the 6 ML SL is an electron-initiated APD, and $\beta$ is close to zero at the lower *M*. In other words, the *F*(*M*) of the 6 ML SL shows that multiplication process is more deterministic, and thus can be extremely low when it operates at the low electric field region where single carrier impact ionization occurs. This result is very promising, and realizing those APDs remains unfinished.

In summary, we report on the engineering of avalanche characteristics in In_0.53_Ga_0.47_As/Al_0.48_In_0.52_As SLs. The InGaAs/AlInAs SLs with the SL periods of 4 ML, 6 ML, and 8 ML were designed to have the same alloy contents as an Al_0.4_Ga_0.07_In_0.53_As RA. The electronic band structures of the SLs computed from the 14-band **K.p** method suggest that the *k*-value of the SLs is lower than that of the RA and become lower as the SL period shortens. The 4 ML SL appears to achieve the lowest *k*-value among them. We also computed the impact ionization rates and coefficients of the RA and the 4 ML SL. The result shows that $\alpha$ of the RA and the 4 ML SL are almost the same while the difference between $\alpha$ and $\beta$ for the 4 ML SL becomes larger than for the RA as the applied electric field decreases. The designed 6 ML and 8 ML SLs are grown by MBE and tested for the verification of the designs. The measured *F*(*M*) of the SLs shows that the 8 ML SL has lower *k*-value (~ 0.22) than the RA, and the 6 ML SL achieved even lower *k*-value than the 8 ML SL, as predicted. The discussion of the design parameters for a low *k*-value SL suggested three design considerations, the position of 1st BZ, the effective mass, and the mini gaps. Further reduction in the *k*-value of the SLs can be achieved by varying M-layer thickness, but the realization of it remains for future research. This work is a theoretical modeling and experimental demonstration of engineering avalanche characteristics in InGaAs/AlInAs SLs and would also assist one to design the SLs with improved performance for various SWIR APD application.

## Methods

The p-i-n SLs with the periods of 6 ML and 8 ML were grown in a RIBER Compact 21DZ molecular beam epitaxy (MBE) chamber on n-type sulfur doped-InP (001) epi-ready substrates. The device structure consists of a 500 nm n^++^-Al_0.48_In_0.52_As bottom contact layer (also used for buffer layer), followed by a 100 nm n^+^-SL for the bottom cladding layer. Subsequently, the M-layer, 1000 nm of unintentionally doped (UID)-SL, was grown to be sandwiched by a 300 nm p^+^-SL top cladding layer and the bottom cladding layer.

**Figure 7:**
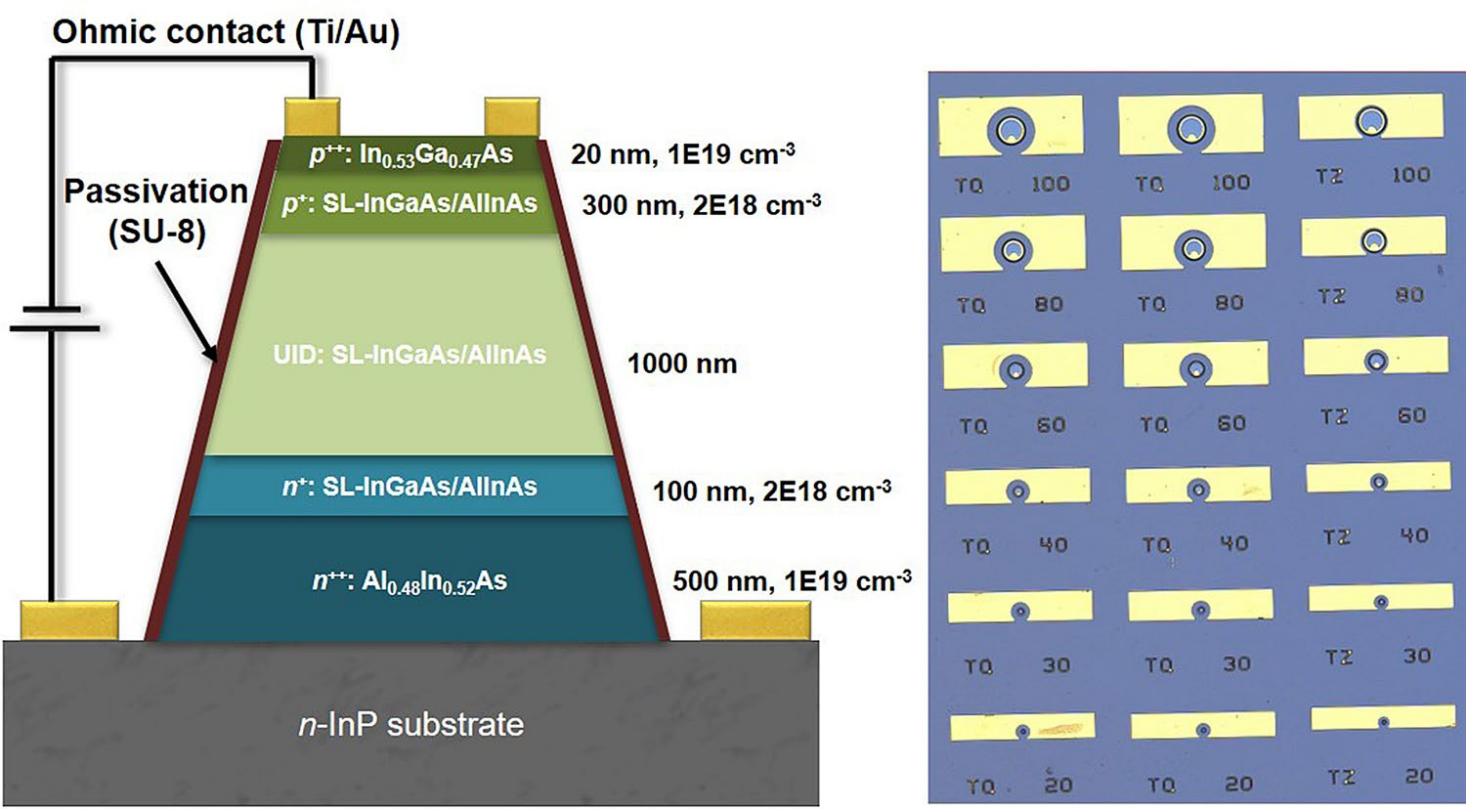
(**a**) Schematic device structure, and (**b**) its top view of microscope image.

It should be noted that the thickness of the top cladding layer is chosen to be thicker than that of the bottom one to ensure a single carrier (electron) injection for proper measurement of the *k*-value. Finally, a 20 nm p^++^-In_0.53_Ga_0.47_As top contact layer was deposited on top of the device structure to ensure an ohmic contact. A detailed diagram of the epitaxial device structure appears in Fig. 7a.

Single-pixel devices, as shown in Fig. 7b, were made with a 4-steps fabrication procedure. Prior to step 1, the grown SL samples were cleaned with acetone, methanol, and isopropyl alcohol in an ultrasonic bath. In step 1, photolithography was performed with a bilayer photoresist, SPR 220 and LOR5A, to open small features for top contact metal deposition. The SL samples were loaded in an oxygen plasma asher to remove leftover resist from the opened areas. The SL samples were then treated with HCl solution to remove the oxidized top layer before loading each into an electron beam evaporator for Ti (20 nm)/Au (150 nm) deposition. In step 2, the second round of photolithography was performed with SPR 220 resist to open areas for etching. A wet chemical etch solution, citric acid (40 g)/H_3_PO_4_ (10 mL)/H_2_O_2_ (10 mL)/H_2_O (240 mL), was used to form the mesa. Step 3 repeated step 1 to form the bottom contact. In step 4, the SL sample was treated with HCl solution to remove the oxide layer on the mesa sidewalls before SU-8 passivation.

**Figure 8:**
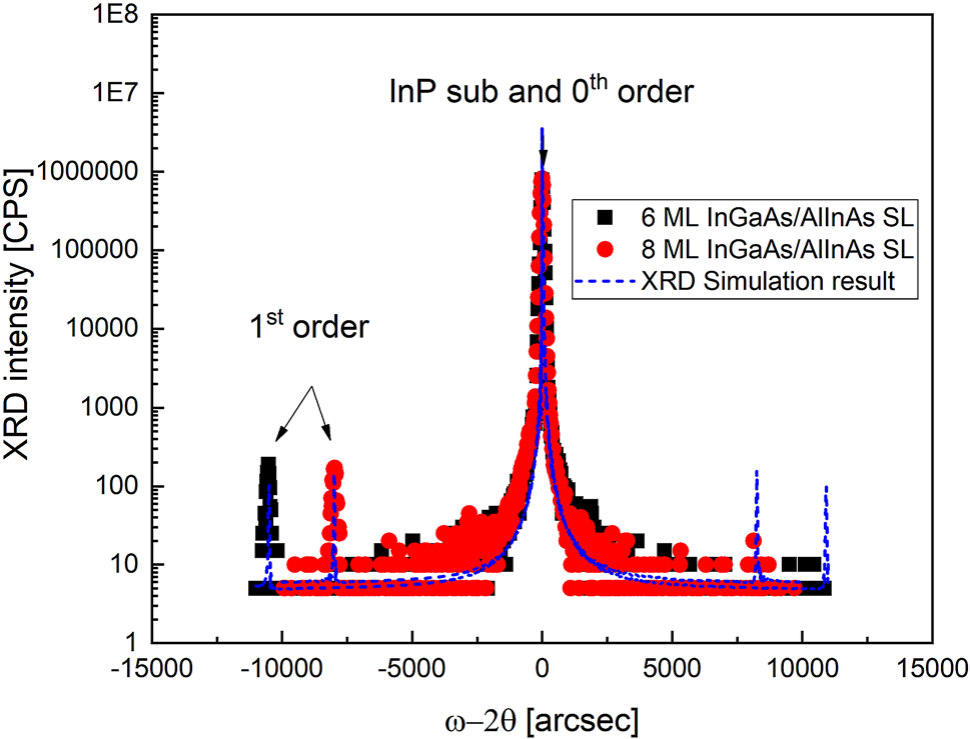
XRD rocking curves for InGaAs/AlInAs SLs with periods of 6 ML and 8 ML.

Prior to the growth of p-i-n SLs, the thickness and composition of each SL constituent ternary alloy were accurately calibrated using reflection high electron energy diffraction (RHEED) and x-ray diffraction (XRD), respectively. Figure 8 shows XRD results of the 6 ML and 8 ML SLs. It is seen that only one peak is found near 0 arcsec, indicating that all layers, including the substrate, buffer, and the SL layers, are perfectly lattice-matched. The periods of the SLs were calculated from a distance between 0^th^ and 1^st^ order satellite peaks and are almost perfectly matched to the designed values described in Table 1.

For the *F*(*M*) measurement, the devices were biased with a Keithley 2400 source meter. A 543 nm He–Ne laser was used to illuminate the APDs to ensure optical absorption near the device surface and provide near-pure electron injection profile for the 6 ML and 8 ML SLs. A standard RF Bias-Tee was used to isolate different signals in the system: the AC component of the output current was measured with an Agilent 8973A noise figure analyzer while still supplying a DC bias to the devices. The noise figure analyzer was calibrated via an Agilent 346A noise source to remove background noise.
